# Toward the future of psychiatric diagnosis: the seven pillars of RDoC

**DOI:** 10.1186/1741-7015-11-126

**Published:** 2013-05-14

**Authors:** Bruce N Cuthbert, Thomas R Insel

**Affiliations:** 1 National Institute of Mental Health, National Institutes of Health, 6001 Executive Boulevard, MSC 9632, Room 7121, Bethesda, MD 20892-9632, USA; 2 National Institute of Mental Health, National Institutes of Health, 6001 Executive Boulevard, Room 8129, Bethesda, MD 20892, USA; 3 Express/parcel delivery, Rockville, MD 20852, USA

**Keywords:** Diagnosis, DSM, ICD, Psychiatric diagnosis, Psychopathology, RDoC, Research domain criteria

## Abstract

**Background:**

Current diagnostic systems for mental disorders rely upon presenting signs and symptoms, with the result that current definitions do not adequately reflect relevant neurobiological and behavioral systems - impeding not only research on etiology and pathophysiology but also the development of new treatments.

**Discussion:**

The National Institute of Mental Health began the Research Domain Criteria (RDoC) project in 2009 to develop a research classification system for mental disorders based upon dimensions of neurobiology and observable behavior. RDoC supports research to explicate fundamental biobehavioral dimensions that cut across current heterogeneous disorder categories. We summarize the rationale, status and long-term goals of RDoC, outline challenges in developing a research classification system (such as construct validity and a suitable process for updating the framework) and discuss seven distinct differences in conception and emphasis from current psychiatric nosologies.

**Summary:**

Future diagnostic systems cannot reflect ongoing advances in genetics, neuroscience and cognitive science until a literature organized around these disciplines is available to inform the revision efforts. The goal of the RDoC project is to provide a framework for research to transform the approach to the nosology of mental disorders.

## Background

As of this writing, there are three versions of diagnostic systems for psychiatry in development. By far the most notoriety has been attached to the revision of the Diagnostic and Statistical Manual of Mental Disorders (DSM) published by the American Psychiatric Association, which has been under revision sufficiently long enough to receive a name change from DSM-V to DSM-5. This attention is not surprising given the prominence of the DSM for clinical diagnosis both in the US and internationally, its simultaneous role in research, and the number of controversial issues that have been involved in the revision process - such as the debates over autism spectrum disorder [1], bereavement and depression [2], and personality disorders [3,4], to name just a few. The DSM revisions have also prompted an extensive re-visiting of important issues regarding the nature of mental disorders, and how they should be considered scientifically. An excellent summary and analysis of these topics is represented by the series of papers that appeared recently in *Philosophy, Ethics, and Humanities in Medicine* and *BMC Medicine* ([5-7]; see also [8]).

The second major revision is that of the Mental and Behavioural Disorders section of the International Classification of Diseases (ICD-11), being developed by the World Health Organization. This revision effort is being accomplished by an international group of experts, including some intentional overlap with members from the DSM committees. (It is worth noting that the ICD represents the official diagnostic standard in the US as in the rest of the world.) Although both the DSM and ICD emphasize clinical utility, the scope of the clinical settings where the ICD is employed tends to be yet more varied and extensive than that of the DSM. The latter is intended largely for use by highly trained mental health professionals (though it is employed by many professional groups). By contrast, the ICD is necessarily designed for health settings around the world, to be used not only by practitioners with widely divergent levels of expertise but also in cultural settings where assumptions about the etiology and nature of disorders may be highly dissimilar from the Western milieu of the DSM. Accordingly, the ICD places stronger emphasis on public health applications than the DSM, and one reflection of this emphasis is the use of definitions that emphasize short text descriptions of each disorder rather than the polythetic symptom lists of the DSM.

Finally, the National Institute of Mental Health (NIMH) instituted the Research Domain Criteria (RDoC) project in early 2009. Given its status as a research classification system rather than one intended for routine clinical use, this initiative diverges markedly from the others in multiple respects. The seven major differences between RDoC and the established systems are delineated in the sections that follow, as its share of this forum.

One caveat is in order at the outset to provide an appropriate context for the remarks that follow. The dictionary reminds us that the first sense of the noun ‘debate’ is ‘a discussion … involving opposing viewpoints,’ as appropriate for its Latin root that means ‘to beat’ [9]. However, discussions among the framers of the DSM-5, the ICD-11 revisions and the NIMH RDoC have from their inception been cordial, and marked by general agreement about the relative emphasis of each respective system and also about their shared interests. Thus - unfortunately from the perspective of sparking a sharp exchange among divergent views - the ‘debate’ in this case must proceed more along the lines of the term’s more elaborated definition, a ‘deliberation’ or ‘consideration.’ In this more congenial sense, there is indeed much to consider.

## Discussion

A diagnostic system can have many purposes. For instance, a major reason for the creation of the ICD was to establish a comprehensive manual for determining causes of mortality, thus enhancing efforts at improving public health. However, perhaps the pre-eminent role of diagnosis in medicine is to determine the exact nature of a patient’s disease in order to administer the optimal treatment. Yet, very little discussion of this aspect can be found either in published papers or in the extensive ‘blogosphere’ that has sprung up around the DSM-5. The revisions have renewed debates about the definition and nature of mental disorders; the various positions in the philosophy of science that might represent how to think about mental illness (‘realist,’ ‘essentialist,’ and so on); categorical versus dimensional approaches to disorders; and the role of reductionism and phenomenology [5-8]. Any discussion about the ramifications of these various considerations in actually making a difference on how we treat our patients, however, has been conspicuously lacking.

This lack is likely due in no small part to the current nature of treatments for mental disorders. On the one hand, effective treatments exist. Treatments for major classes of disorders such as depression, anxiety disorders, schizophrenia and bipolar disorders are available, and effective for large numbers of patients. Further, a number of effective treatment modalities - pharmaceutical interventions, psychosocial or behavioral treatments, medical devices - have been established. On the other hand, treatments are not particularly precise, and tend to affect broad classes of disorders. Anti-depressant medications, such as selective serotonin reuptake inhibitors, are used to treat not only depression but a wide variety of anxiety, mood and other disorders. Anti-psychotic agents are used not only with schizophrenia but in bipolar disorder and sometimes for personality and other severe disorders. Anxiolytics such as valium are prescribed widely across the anxiety and mood spectrum. A similar situation prevails for behavioral treatments; for instance, the use of cognitive-behavioral therapy, albeit with many variants, has expanded beyond the internalizing disorders spectrum for which it was originally developed to the treatment of virtually all mental disorders (for example, see [10]).

Although decent treatments for mental disorders are thus plentiful, it is instructive to contrast the changes in disease burden for other diseases over the past several decades with that for mental disorders. For instance, the impact of research - both clinically and in public health arenas - has been dramatic for heart disease. Death due to heart disease climbed steadily from 1950 through 1968, at a rate that projected almost 1.8 million deaths in 2007. Instead, because of the rapid progress of research, the actual mortality due to heart disease was only about one quarter of that number; approximately 1.1 million deaths in 2007 alone were averted according to the predicted peak rate [11]. Similarly, survival rates for children with acute lymphoblastic leukemia have improved over the last several decades from less than 10% to over 90% [12]. By contrast, mortality has not decreased for any mental illness, prevalence rates are similarly unchanged [13], there are no clinical tests for diagnosis, detection of disorders is delayed well beyond generally accepted onset of pathology, and there are no well-developed preventive interventions.

There are many reasons for this lack of progress in mental disorders. The brain is the most complex organ in the body, and it is well-accepted that mental illnesses involve highly complex interactions of genetic factors and experience. The brain cannot be studied directly with the facility we have for more accessible organs, limiting progress based on pathology. However, the diagnostic system for psychiatry has also been increasingly noted as an impediment to progress. The problems have been extensively documented (for example, [14-18]) and do not need to be elaborated here, but include excessive co-morbidity of disorders, marked heterogeneity of mechanisms and reification of disorders. In particular, the underlying validity of the disease entities has been questioned, in that the DSM and ICD categories do not map well onto emerging findings from genetics, systems neuroscience and behavioral science (for example, [19,20]); as a result, it becomes very difficult to translate research from basic studies, either in animal models or in humans, to a systematic understanding of pathology or to systematic treatments directed at mechanisms. Nevertheless, the DSM and ICD system (the two nosologies are largely overlapping in terms of the actual listing of disorders) has become the standard to obtain research grants regarding etiology and pathophysiology, to conduct drug trials at all phases, and to obtain regulatory approvals for pharmaceutical treatments. In behavioral research as well, the need to establish evidence-based treatments has led researchers to copy the lead of drug trials and conduct trials in terms of DSM and ICD diagnoses. Thus, issues with the current nosology markedly affect the treatment development arena.

This point is well illustrated in a quotation from a recent paper by several pharmaceutical industry scientists regarding problems in drug development using the current system: ‘On average, a marketed psychiatric drug is efficacious in approximately half of the patients who take it. One reason for this low response rate is the artificial grouping of heterogeneous syndromes with different pathophysiological mechanisms into one disorder … by increasing the mechanistic understanding of disease and matching the right treatments to the right patients, one could move from one-size-fits-all to targeted therapy and increase the benefit-risk ratio for patients.’ These scientists conclude that a ‘pedestrian’ trial-and-error search of multi-target agents must be hazarded ‘… until clinical trial design and patient segmentation can improve to the point of matching disease phenotype to circuit-based deficits…’ ([21], p. 1276). This problem is no doubt a not insignificant reason why so many pharmaceutical companies have withdrawn from active development research in mental disorders [22,23]. And the reliance on biologically heterogeneous categories as the gold standard for diagnosis has clearly precluded the identification or validation of biomarkers. Although one could imagine revising the diagnostic categories to align with biological discoveries, our field has essentially excluded biological findings that do not map on to the current heterogeneous categories of symptom clusters.

In other areas of medicine, trends have increasingly moved in the direction of ever more precise specification of the genetic, molecular and cellular aspects of disease. In specialty after specialty, there has been a realization that disease entities that appear to be a single disorder actually have distinct genetic precursors and pathophysiology. For instance, for many forms of cancer, diagnosis is no longer defined by the involved organ or even the pathologist’s report, but rather by analysis of genetic variants that can predict exactly what treatment will be optimal (for example, [24]). In another domain, perhaps the most striking example of this trend involves a new drug, Ivacaftor (Kalydeco), approved by the Food and Drug Administration after an expedited review. The drug is effective in treating patients with cystic fibrosis who have a form of the syndrome with a specific mutation of the cystic fibrosis transmembrane regulator gene. Only 4% of patients with cystic fibrosis have this genetic mutation, but, for these patients, the compound is highly effective in correcting the action of the malfunctioning protein [25].

These new approaches toward individualized treatment are now generally called ‘precision medicine’, and represent the forefront of medical science. In November 2011, the US National Academy of Sciences published a major report on precision medicine outlining the significance of this development and calling for new knowledge networks that can harness the power of promising technologies to identify and correct specific pathophysiologies that result from genetic and environmental causes [26]. As yet, the field of mental disorders research lags badly behind the rest of medicine in moving toward precision medicine; yet, knowledge of the central nervous system has exploded over the last two decades, and new technologies are rapidly eclipsing such well-known methods as positron emission tomography scans and magnetic resonance imaging. How can these rapid developments in basic science be harnessed in the service of precision medicine for mental disorders?

### Research domain criteria

As a national health ministry, NIMH is committed to reducing the burden of suffering due to mental illness through research. Decades of research have increasingly revealed that neural circuits and systems are a critical factor in how the brain is organized and functions, and how genetics and epigenetics exert their influence. However, this knowledge cannot be implemented in clinical studies as readily as might be hoped. Any one mechanism, such as fear circuits or working memory, is implicated in multiple disorders as currently defined; it is difficult to know which diagnostic category to select first to explore any promising leads, and a positive result immediately raises the question of whether the demonstration of efficacy must be extended to all similar disorders (a time-consuming and expensive proposition). Contrariwise, a syndrome such as major depression clearly involves multiple mechanisms - dysfunction in the hypothalamic pituitary axis, in brain reward-seeking activities, in emotion regulation circuits, in modulatory neurotransmitter systems, in cognitive systems, and in epigenetic marks; thus, it is not surprising that studies to establish ‘the cause’ of major depression are equivocal and difficult to replicate, nor that new treatments directed toward a particular mechanism are often only marginally effective and cannot be replicated.

In response to this situation, NIMH established in its Strategic Plan of 2008 the following goal: to ‘develop, for research purposes, new ways of classifying mental disorders based on dimensions of observable behavior and neurobiological measures.’ The instantiation of this goal is the RDoC project, and is NIMH’s effort to develop a precision medicine approach for mental disorders [27].

RDoC represents a real paradigm shift, by considering mental disorders from a translational point of view. RDoC does not take as a starting point the traditional view of disorders as symptom complexes based largely on clinical descriptions. Rather, the approach proceeds in two steps. The first step is to inventory the fundamental, primary behavioral functions that the brain has evolved to carry out, and to specify the neural systems that are primarily responsible for implementing these functions. For instance, much is now known about circuits for fear and defense [28], for various aspects of appetitive behavior such as learning to predict reward and moving toward reward [29], and cognitive functions such as working memory [30]. The second step then involves a consideration of psychopathology in terms of dysfunction of various kinds and degrees in particular systems, as studied from an integrative, multi-systems point of view.

The four aims of the RDoC project are listed in Table 1, beneath the statement of goal 1.4. The project began with deliberations among members of an internal NIMH working group, which served to define the overall shape of the effort as well as the specific process to be followed. The workgroup determined that the optimal approach was to establish a hierarchical scheme, with the specific dimensions nested within five major domains of functioning (see Table 2 for a listing of the RDoC matrix as of June, 2012 at the end of the initial conference series). The project moved forward rapidly once this organizational matrix was established. As called for Aim 1 of Table 1, the RDoC process involved a series of workshops with experts in the field to determine the ‘fundamental behavioral components’ to be included in the system. The five major domains, conceived on empirical grounds from such diverse research areas as temperament, behavior genetics and structural models of mental disorders, also served as a convenient way to organize the workshops in that one workshop was conducted for each of the five domains. Approximately 30 to 40 experts convened for each workshop. Their charge was to determine which dimensions should be included within the domain; provide a definition for each dimension; and provide a list of the elements for each dimension that could be used to measure it, at each of several units of analysis (as specified in Aim 4 of Table 1). An important consideration is that the dimensions, as behavioral entities tied to neural systems, are always dependent upon the march of research to continually refine and evolve a scientific understanding of their function and of their implementing circuits. In this sense, the dimensions represent ‘constructs’ as classically defined in psychological research [31], and this term was adopted for RDoC to emphasize that they will (and should) always be subject to further validation and revision. The RDoC ‘matrix’ thus consists of a series of rows, with the constructs nested within their superordinate domains, and the columns representing the units of analysis. The reader is encouraged to consult the RDoC website (http://www.nimh.nih.gov/research-funding/rdoc/index.shtml), which contains the completed matrices from all of the RDoC workshops.

**Table 1:** National Institute of Mental Health Strategic Goal 1.4: Develop, for research purposes, new ways of classifying mental disorders based on dimensions of observable behavior and neurobiological measures

**Aim #**	**Task**
1	Initiate a process for bringing together experts in clinical and basic sciences to jointly identify the fundamental behavioral components that may span multiple disorders (e.g., executive functioning, affect regulation, person perception) and that are more amenable to neuroscience approaches.
2	Determine the full range of variation, from normal to abnormal, among the fundamental components to improve understanding of what is typical versus pathological.
3	Develop reliable and valid measures of these fundamental components of mental disorders for use in basic studies and in more clinical settings.
4	Integrate the fundamental genetic, neurobiological, behavioral, environmental, and experiential components that comprise these mental disorders.

**Table 2:** Research domain criteria, October 2012 (constructs are listed within each domain)

**Negative valence domain**	**Positive valence systems**	**Cognitive systems**	**Systems for social processes**	**Arousal/modulatory systems**
Acute threat (‘fear’)	Approach motivation	Attention	Affiliation and attachment	Arousal
Potential threat (‘anxiety’)	Initial responsiveness to reward	Perception	Social communication	Biological rhythms
Sustained threat	Sustained responsiveness to reward	Working memory	Perception and understanding of self	Sleep-wake
Loss	Reward learning	Declarative memory	Perception and understanding of others	
Frustrative nonreward	Habit	Language behavior		
		Cognitive (effortful) control		

### The seven pillars

The distinctions between RDoC and the DSM and ICD systems can be captured by seven major points that include both conceptual and practical differences. First, the approach incorporates a strong translational research perspective. Rather than starting with symptom-based definitions of disorders and working toward their pathophysiology, RDoC inverts this process. Basic science - in genetics, other areas of neuroscience and behavioral science -serves as the starting point, and disorders are considered in terms of disruptions of the normal-range operation of these systems, with an emphasis on the mechanisms that serve to result in dysfunctions of varying degrees.

Second, RDoC incorporates an explicitly dimensional approach to psychopathology, as called for in many recent analyses of psychopathology [32,33]. However, in contrast to views that emphasize dimensionality mostly as a function of symptom severity, RDoC is committed to studying the ‘full range of variation, from normal to abnormal.’ In some cases, only one end of a dimension may involve problem behavior (for instance, one is seldom likely to complain of an outstanding memory or keen vision), but often both extremes of a dimension may be considered as ‘abnormal’ – for example, a complete lack of fear may be associated with aggressive or psychopathic behavior, and the opposite end of diminished reward-seeking may be mania. An important consideration regarding dimensionality is that the relationship between increasing disruptions in functional mechanisms and the severity of symptoms may be markedly nonlinear, with ‘tipping points’ that mark a transition to more severe pathology; a critical area of research is to determine the exact location of such points, and how they are affected in each individual by various risk or resilience factors.

The third distinction follows directly from the second. Aim 3 in Table 1 includes a call to ‘Develop reliable and valid measures of these fundamental components.’ One of the drawbacks of a pathogen model of illness is that most scales developed over the past decades have either been designed to study normal traits such as personality or else clinical symptoms of disorder, and thus lack sensitivity at one end or the other of a putative dimension. In particular, zones of very mild or transient psychopathology, with their potential for understanding proximate etiology and for indicated prevention, receive short shrift. Thus, scale development represents a high priority for RDoC research applications. In fact, well-validated and psychometrically optimized measures based upon cognitive neuroscience research are beginning to appear [34]. Consistent with contemporary measurement science, new scales would (and should) almost invariably incorporate interval or ratio scaling to improve quantification of the phenomena of interest. As such assessments muster, it becomes feasible to determine cut-points along the distribution for varying types of interventions, essentially similar to practices in other areas of medicine where continuous measures are available, such as hypertension or hypercholesterolemia. A further advantage of this approach is that ongoing research studies about relative risk at various points along the dimension can inform decisions about changing the cut-points at which interventions are indicated - as has happened repeatedly, such as in hypertension research [35].

The fourth distinction concerns the types of designs and sampling strategies that RDoC studies must necessarily follow. In the traditional clinical study, the independent variable is almost always one or more (usually one) DSM or ICD groups, often versus controls. It is relatively straightforward to diagnose the patients according to the symptom-based criteria, excluding those who fail to meet criteria for the diagnosis under study. The resultant groups form the independent (grouping) variable. (An important public health issue concerns the unknown number of such patients whose conditions are essentially invisible to research by virtue of failing to meet criteria, although it is well known that for some disorders, such as eating disorders, ‘not otherwise specified’ is the modal diagnosis.) RDoC, by contrast, involves a two-step procedure. The investigator must first establish the ‘sampling frame,’ that is, what group of individuals will be entered into the study; because this will not be identical to a DSM or ICD diagnosis, other criteria will have to be applied. In some cases, this might simply comprise all patients presenting at a certain type of clinic, such as for anxiety disorders or serious mental illness. However, such a sampling frame might fail to meet the goal of studying the ‘full range,’ and so a control group might also be needed - with a wider range of inclusion, however, rather than the typical ‘super-normal’ control group with no psychiatric history. Then, the second step is to specify the independent variable in the study. To permit investigators freedom in pursuing their hypotheses, the independent variable may be chosen from any unit of analysis. Thus, performance on a working memory task might be the independent variable for a study of working memory in serious mental illness; dependent variables might comprise neuroimaging of specified brain areas, relevant assessments of real-world dysfunction and an exploration of relevant candidate genes. For a study of anxiety disorders, fear-potentiated startle might be the independent variable, stratified by a relevant genetic polymorphism, and the dependent variables could be overall symptom severity and distress plus performance on a behavioral fear-avoidance test. Thus, while more interesting research designs can be created, the investigator will need to be more thoughtful about crafting the design of the study to answer the particular experimental question.

Fifth, and critically important, the system is intended to provide a structure that places equal weight on behavioral functions and upon neural circuits and their constituent elements - that is, to be an integrative model rather than one based primarily on either behavior or neuroscience. This integrative approach can be seen in the way in which goal 1.4 is stated. The criterion for including a construct in the matrix during the workshops reflects this same priority. Participants were instructed that there were two requirements for adding a construct to the matrix: first, ‘There must be strong evidence for the validity of the suggested construct itself [as a behavioral function]’; second, ‘There must be strong evidence that the suggested construct maps onto a specific biological system, such as a brain circuit.’ This rule was carefully followed; over the course of the workshop series, there were several instances where a nominated construct was not included either because a nominated function could not be paired with an implementing neural system, or because a consensus could not be reached regarding the function of a nominated circuit. The NIMH working group’s shorthand expression for this idea was, ‘Behavioral science studies what the brain evolved to do, and neuroscience studies how the brain implements it.’ Thus, claims that the RDoC system simply involves biomarkers or endophenotypes are over-simplified at best.

Following from this consideration, a sixth distinction is that the RDoC project is intended (at its inception, in particular) to concentrate on constructs for which there is solid evidence to serve as a platform for ongoing research. There is no claim to include all of the psychopathology that is listed in the various categories of the DSM and ICD nosologies. This reflects a deliberate decision by NIMH to constrain the initial scope of the project to elements for which there is considerable data, so as to provide a solid foundation on which to gain experience and indicate how more provisional constructs may be studied profitably in the future.

Finally, a research-oriented scheme like RDoC faces both a luxury and a risk in not being tied to fixed definitions of disorders. As many commentators have pointed out, any changes to DSM or ICD criteria prompt considerable upheaval throughout the mental health system - in officially reported prevalence rates, in possible insurance reimbursement changes, in legal proceedings and declarations of disability, in regulatory practice. As an experimental classification, RDoC does not face these liabilities. In fact, a strong goal of a research system ought to be its flexibility in dynamically accommodating those research advances that it tries to foster. Provision must be made to delete constructs that have been superseded by new thinking, to add constructs, to split one construct into two, and so on. (The NIMH RDoC workgroup has actively considered the optimal process for considering such changes, which will be disseminated in the near future.) As this consideration implies, and in contrast to clinical nosologies, the constructs appearing in the RDoC matrix (Table 2) are not the only ones that can be studied. A new construct can be added to the matrix only when replicated data are furnished to provide evidence that it meets the two criteria indicated above (a validated construct, and a specifiable neural circuit); it follows that such studies could not be conducted if only those constructs listed in the RDoC matrix were permitted for study. Thus, a critical component of RDoC is to permit research involving well-justified experiments seeking to validate constructs that are not currently part of the RDoC matrix, or to modify in various ways the extant constructs.

## Summary

Psychiatry lags behind other areas of medicine in building avenues toward a precision medicine approach to diagnosis, and will not catch up until a system is available that reflects recent progress in genetics, other areas of neuroscience and behavioral science. However, such a system cannot be implemented until a database is available that can inform its development. This is the essential rationale for the RDoC project. It is difficult to estimate how long such a project may take. Already, promising developments are being forged by investigators who have probed the circuits from both basic and clinical directions, and have related these findings to well-validated tasks that measure functioning. However, the integrative approach that RDoC calls for is so new that unforeseen obstacles surely await the pioneers in this area. This is only to be expected. In the long run, there seems to be a growing consensus in the field that a more empirically based approach must be developed, and the inherent qualities of the research process itself should serve to shape mid-course corrections as the project moves forward. It should be re-iterated, however, that the RDoC framework is explicitly intended to be a moving target, and that the framework should grow and change with the pace of new research findings. Thus, the challenge is not to design an optimal list of relatively permanent elements, but rather to construct a platform that can both accommodate and foster continual developments in research knowledge and methods.

It will be quite apparent to the reader that RDoC is neither designed nor intended to be used for practical clinical purposes at this early stage. The near-term goal of RDoC, rather, is to build a new *framework* of research that can produce pioneering new findings and approaches to inform future versions of psychiatric nosologies. In particular, the goal is to lay the groundwork for specifying how diagnosticians can accomplish the goal of precision medicine for mental disorders - pinpointing with increasing accuracy the precise genetic, neural circuit and behavioral data that can generate tailored recommendations for interventions that can manage, cure and prevent mental disorders in the largest possible number of individuals. In this sense, although the immediate thrust of the RDoC project sets it apart from the established structures of the DSM and ICD, the long-term aspirations for all three systems converge on reducing the burden of suffering for those with mental disorders.

## Abbreviations

DSM: Diagnostic and Statistical Manual of Mental Disorders; ICD: International Classification of Diseases; NIMH: National Institute of Mental Health; RDoC: Research Domain Criteria

## Competing interests

The authors declare that they have no competing interests.

## Authors’ contributions

Both authors have discussed the ideas expressed herein, and contributed to final versions of the paper. BC provided the first draft and final draft based upon comments from TI and discussions between both authors. Both authors read and approved the final manuscript.

## Pre-publication history

The pre-publication history for this paper can be accessed here:

http://www.biomedcentral.com/1741-7015/11/126/prepub
